# Prenatal phthalate exposure and sex steroid hormones in newborns: Taiwan Maternal and Infant Cohort Study

**DOI:** 10.1371/journal.pone.0297631

**Published:** 2024-03-14

**Authors:** Chin-Li Lu, Hui-Ju Wen, Mei-Lien Chen, Chien-Wen Sun, Chia-Jung Hsieh, Ming-Tsang Wu, Shu-Li Wang, TMICS study group

**Affiliations:** 1 Graduate Institute of Food Safety, College of Agriculture and Natural Resources, National Chung Hsing University, Taichung, Taiwan; 2 Department of Food Science and Biotechnology, College of Agriculture and Natural Resources, National Chung Hsing University, Taichung, Taiwan; 3 Department of Post-Baccalaureate Medicine, College of Medicine, National Chung Hsing University, Taichung, Taiwan; 4 National Institute of Environmental Health Sciences, National Health Research Institutes, Miaoli, Taiwan; 5 Institute of Environmental and Occupational Health Sciences, College of Medicine, National Yang Ming Chiao Tung University, Taipei, Taiwan; 6 Department of Public Health, Tzu Chi University, Hualien, Taiwan; 7 Graduate Institute of Clinical Medicine, Kaohsiung Medical University, Kaohsiung, Taiwan; 8 Department of Safety, Health, and Environmental Engineering, National United University, Miaoli, Taiwan; King Faisal Specialist Hospital and Research Center, SAUDI ARABIA

## Abstract

**Background:**

Newborn anogenital distance (AGD) has been associated with prenatal exposure of phthalates. The association between prenatal phthalate exposure and sex steroid hormones in newborns is unclear.

**Object:**

This study aimed to examine whether cord-blood sex hormone levels were associated with prenatal phthalate exposure and newborn anogenital distance (AGD).

**Methods:**

In the Taiwan Maternal and Infant Cohort Study, we recruited 1,676 pregnant women in their third trimester in 2012–2015 in Taiwan. We determined 11 urinary phthalate metabolites in pregnant women, three maternal and five cord-blood steroid sex-hormone concentrations. Five hundred and sixty-five mother-infant pairs with sufficient data were included. Trained neonatologists measured 263 newborns’ AGD. We examined the associations of prenatal phthalate metabolite levels with AGD and hormones using linear regression models and evaluated correlations between maternal and cord-blood sex hormone levels and AGD.

**Results:**

Compared with the male newborns exposed to maternal phthalate metabolites at the first tertile, AGD was -3.75, -3.43, and -3.53 mm shorter among those exposed at the median tertile of di-2-ethylhexyl phthalate (DEHP) metabolites, monobenzyl phthalate (MBzP), and monomethyl phthalate (MMP), respectively. Compared with those who had exposed at the first tertile, cord-blood follicle-stimulating hormone (FSH) decreased among male newborns exposed at higher levels of MMP, mono-n-butyl phthalate (MnBP), MBzP and DEHP, and among female newborns exposed at higher levels of MMP, MBzP and mono(2-ethyl-5-hydroxyhexyl) phthalate. However, we did not observe significant correlations of maternal or cord-blood sex steroid hormones with newborns’ AGDs.

**Conclusions:**

Alterations in cord-blood sex steroid hormone levels were associated with prenatal phthalate exposures, particularly in male newborns. Women aspiring to be pregnant should be alerted of the need of reducing phthalate exposure.

## Introduction

Phthalate esters comprise a group of chemicals found in products widely used in our daily life, such as plasticizers in polyvinyl chloride, fixatives in personal care products (PCPs), and adhesives in building materials [1]. In addition to dermal absorption and inhalation, oral ingestion of contaminated food is a major phthalate entry route of (i.e., di-2-ethylhexyl phthalate [DEHP]) into the human body [2]. Once environmental phthalates enter human body, they are rapidly hydrolyzed to monoesters and oxidized compounds.

The parent compounds and metabolites of phthalates can penetrate placenta and enter fetal circulation [3], and mimic or block the transcriptional activation of steroid hormones [4]. Cord-blood levels of sex steroid hormones are related to both hormones secreted by placenta and the circulating hormones in fetus, which potentially influence fetus’ growth [5]. Investigating the effects of prenatal phthalate exposure on cord-blood sex hormones and neonatal reproductive system may further advance our understanding of the reproductive toxicity of phthalates.

In March 2011, a phthalate-tainted-foodstuff episode occurred in Taiwan. Broad food items were contaminated by fake cloudy agents containing phthalates and successively withdrawn from the shelves. After the episode, the regulations in Taiwan, consistent to EU and US, continuously restrict the use of phthalates and setting concentration limits in commonly used phthalates in toys and child care articles, food contact materials, and cosmetics [6]. Subsequent studies found that the urinary levels of phthalate metabolites in pregnant women had significantly decreased several years later [7, 8]. Nowadays, it comes to the question of whether the offspring would be exempted from disturbance on reproductive and endocrine system under the relatively low prenatal phthalate exposure in Taiwan. Despite numerous evidences have shown an inverse association between prenatal exposure of phthalates and anogenital distance (AGD) in male newborns [9–11], evidences in female newborns are rare. Moreover, to the best of our knowledge, only two epidemiological studies have assessed the effects of maternal phthalates exposure on fetal sex steroid hormones in birth cohorts, and the findings were inconclusive [12, 13].

AGD is a sensitive marker to detect anti-androgen effect in newborns, it depends on in-utero testosterone secreted from embryonic testicle from the early stage of pregnancy [14]. Experimental studies have shown reproductive malformations and fertility reductions in male offspring of pregnant rats co-administered with dibutyl phthalate and DEHP [15]. Epidemiological studies have also found a decrease in AGD in male infants associated with higher maternal monoethylhexyl phthalate (MEHP), monobenzyl phthalate (MBzP), and mono-n-butyl phthalate (MnBP) [9–11]. Prenatal exposure to phthalates may have sex-specific effects on reproductive organ development in male and female offspring. However, the adverse effects of phthalates on female reproductive outcomes have been less reported and inconclusive [16].

Numerous epidemiologic studies based on cross-sectional design had reported phthalate exposures are associated with sex steroid hormone levels [17]. However, only a few studies investigated the impact of prenatal exposure of phthalates and offspring’s sex steroid hormones [16]. Among the rarely available literatures, the Hokkaido Cohort Study (HCS) only investigated MEHP levels in maternal serum and found that the effect of phthalates was more pronounced in male newborns [12, 18]. Lin et al. only reported phthalate effects on cord-blood estradiol (E2), free testosterone (frT) levels and frT/E2 ratio and observed significant associations only in female newborns [13]. Differences in target hormones, selected phthalates, exposure levels, and sample matrices may contribute to the inconsistency between studies.

Our study analyzed data from the Taiwan Maternal and Infant Cohort Study (TMICS), which enrolled pregnant women after the 2011 phthalate-tainted-foodstuff episode. We aimed to examine the potential impacts of prenatal phthalate exposures on AGD and fetal sex steroid hormones in male and female newborns, respectively, and evaluate the mediation effect of cord-blood sex steroid hormones on the relationship between prenatal phthalate exposures and alterations of AGD at birth.

## Materials and methods

### Study cohort and study design

**Fig 1:**
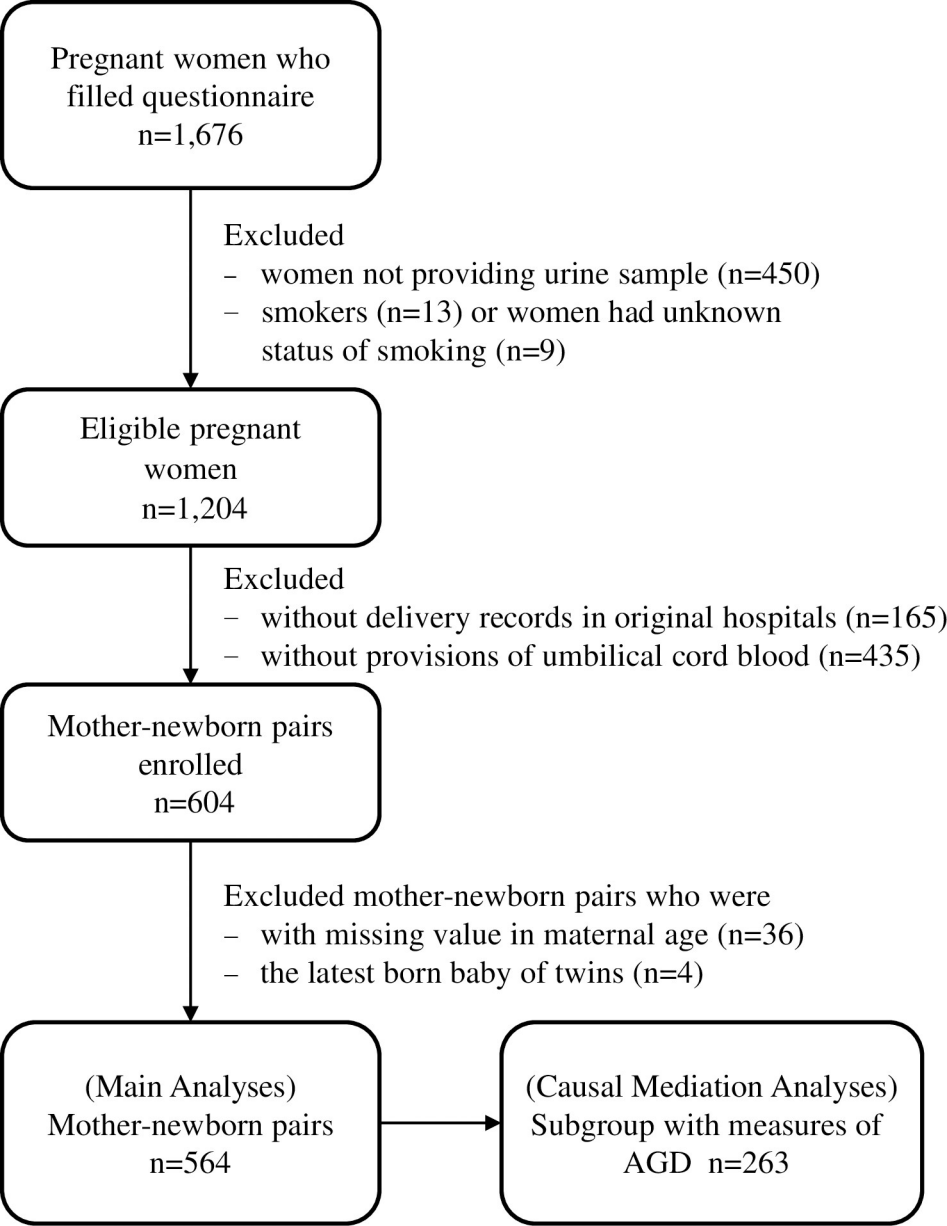
Flow chart of participants enrolled in this study.

Our subjects were from the TMICS, as previously described in detail [7]. In brief, pregnant women in their third trimester at nine hospitals located in northern, central, southern, and eastern Taiwan between October 25, 2012 and May 21, 2015 were invited to participate in the TMICS. Participant inclusion and exclusion criteria are shown in **Fig 1**. Self-administered questionnaires, anthropometric measures, and bio-samples (i.e., urine and peripheral blood) were obtained from participants during the baseline interview. Umbilical cord-blood samples were immediately collected after babies born and stored at –20°C until analysis. The study protocol was approved by the Institutional Review Board of the National Health Research Institute (NHRI) and our nine collaborating hospitals, the full names of the nine IRBs are listed in **S1 Table**. A written informed consent was obtained from each participant.

### Phthalate exposure

One-spot urine samples were collected during pregnancy at outpatient departments, delivered to the central laboratory at the NHRI, and certificated by an international laboratory comparison program (G-EQUAS 59) [7]. The concentrations (μg/L) of eleven urinary phthalate metabolites were determined using a solid-phase extraction method coupled with liquid chromatography-electrospray ionization tandem mass spectrometry (LC-ESI-MS/MS, Agilent 1200/API 4000). The phthalate metabolites we measured included MEP, monomethyl phthalate (MMP), monoisobutyl phthalate (MiBP), monoisononyl phthalate (MiNP), mono-(2-ethyl-5-hydroxylhexyl) phthalate (MEHHP), mono-(2-ethyl-5-oxohexyl) phthalate (MEOHP), mono(2-ethyl-5-carboxypentyl) phthalate (MECPP), and mono(2-carboxymethylhexyl) phthalate (MCMHP) (Hsieh et al. 2019). In brief, we prepared 100 μL urine sample aliquots containing 20 μL ammonium acetate (1 M, pH 6.5), 10 μL β-glucuronidase, and a mixture of isotopic phthalate metabolite standards. The samples were incubated at 37°C for 1.5 h and treated with 270 μL solvent (5% acetonitrile and 0.1% formic acid) in glass screw-cap vials and mixed for quantitative LC-ESI-MS/MS after hydrolysis. For quality control, a blank sample’s measured concentration below two-fold the limit of detection (LOD) value (LOD for MMP, MEP, MnBP, MiBP, MBzP, MiNP, MEHP, MEHHP, and MEOHP was: 0.12, 0.06, 0.06, 0.12, 0.06, 0.6, 0.12, 0.06, 0.12 μg/L, respectively) for urinary metabolites was required. Each phthalate metabolite’s recovery rate ranged from 80% to 119%. Phthalate metabolite concentrations below the LOD were assigned a value of 1/2 LOD.

Considering the urinary dilution effect, urinary phthalate metabolite levels were divided by urinary creatinine levels measured using the ADVIA 1800 Clinical Chemistry System (Siemens, Erlangen, Germany) at the Union Clinical Laboratory (UCL; Taipei, Taiwan). Because MECPP and MCMHP tests were only available for limited study participants, we calculated the micromolar sum of MEHP, MEOHP, and MEHHP levels to estimate DEHP exposure (ΣDEHP).

### Anogenital distances

Among the 564 babies, 263 were assessed for their AGDs by trained neonatologists who had been trained by watching an instructional video. AGD was measured as the scrotum-to-anus distance in male newborns and the distance from the center of the anus to the posterior convergence of the fourchette in female newborns. Babies were kept in a supine position, and their legs were held back in a frog-leg posture to identify the central position of the anus. The basic AGD-measurement procedures followed the methods developed by the Infant Development and Environment Study [19]. Each AGD was measured twice per baby using a dial caliper and the averaged distance was used for data analysis.

### Sex steroid hormone concentrations

Clinical-biochemistry concentration were determined in radioimmunoassay (Diagnostic Products Corporation, Los Angeles, CA, USA) which was performed at UCL. The measures included five cord-blood sex steroid hormones, i.e., progesterone (ng/mL), E2 (pg/mL), frTT (ng/dL), sex hormone-binding globulin (SHBG, nmol/L), and follicle stimulating hormone (FSH, mIU/mL), as well as three maternal sex steroid hormones, i.e. progesterone (ng/mL), E2 (pg/mL), total testosterone (TT, ng/dL). The detection limit values of progesterone, FSH, E2, free TT, and SHBG were 30 ng/mL, 0.3 mIU/mL, 11.8 pg/mL, 0.04 ng/dL, and 0.35 nmol/L, respectively.

### Statistical methods

We used geometric mean with geometric standard deviation and median with interquartile range to describe right-skewed variables and the Kruskal–Wallis H test to compare differences between groups. We categorized phthalate metabolite levels into low, median, and high based on tertile cut-off points for all phthalate metabolites but MBzP. Because of lower detection rate of MBzP, we assigned undetectable MBzP levels as low and dichotomized detectable values as median and high. Linear regression model was performed to estimate the changes of AGD corresponding to exposure levels of phthalate metabolites from low-to-middle and low-to-high levels. According to literature review, basic sociodemographic characteristics (household income, maternal education status), maternal physical activity, birth outcomes, gestational diabetes in pregnancy, and delivery records were considered potential confounders. Finally, we used the directed acyclic graph (DAG) analysis to determine the covariates which should be correctly adjusted in multivariable regression analyses **(S1 Fig).** Because body size and age largely explain variation in AGD, the newborn’s percentile of weight-for-length was included as a covariate [19] in predicting AGD. The norm of weight-for-length in the World Health Organization Growth Standard for babies 0–2 years of age [20] was applied. To explore the potential mediation, we additionally conducted general linear regression models to explore the associations of cord-blood and maternal sex hormone levels with newborn’s AGD in **S5 Table**, the sex hormone levels were in natural logarithm scale in these analyses.

All data analyses were performed using SAS software (version 9.4; SAS Inc., Cary, NC, USA).

## Results

The present study recruited 564 mother-infant pairs from the TMICS (**Fig 1**). **Table 1** shows the maternal urinary concentrations of MMP, MEP, MnBP, MiBP, MBzP, MiNP, and DEHP metabolites in male and female newborns. Phthalate metabolite detection rates were generally high, except for MiNP (4%) and MBzP (46%). We therefore did not include MiNP in subsequent analyses. Maternal concentrations of phthalate metabolites were similar between mothers who initially provided urines at the 3^rd^ trimester and those who finally included in data analyses **(S2 Fig)**. In-utero phthalate metabolite levels differed according to maternal age, education level, household income, exercise frequency, gestational weight gain, and term pregnancy **(S2 Table)**.

**Table 1:** Prenatal exposure of phthalate metabolites in female and male newborns.

Parent compound	Phthalate Metabolite	Female newborns (N = 270)			Male newborns (N = 294)			*P*-value
	(μg/ g creatinine)	DR %	GM (GSD)	Median (Q1-Q3)	DR %	GM (GSD)	Median (Q1-Q3)	
Di-methyl phthalate (DMP)	MMP	94.8	6.89 (3.44)	7.79 (4.14–14.26)	93.5	7.52 (3.90)	8.95 (4.89–15.79)	0.095
Diethyl phthalate (DEP)	MEP	89.6	15.85 (7.35)	19.22 (7.85–55.48)	89.8	16.54 (7.69)	19.41 (7.75–54.77)	0.992
Di-n-butyl phthalate (DnBP)	MnBP	99.6	23.13 (2.40)	22.53 (13.58–35.82)	98.3	21.84 (2.70)	22.31 (13.26–35.7)	0.764
Di-isobutyl phthalate (DiBP)	MiBP	97.0	11.49 (2.59)	11.77 (6.85–21.09)	97.6	11.41 (2.39)	11.29 (7.26–18.65)	0.821
Benzyl butyl phthalate (BBzP)	MBzP	46.3	0.46 (5.12)	0.60 (0.60–1.42)	46.3	0.51 (5.66)	0.60 (0.60–1.71)	0.540
Di-isononyl phthalate (DINP)	MiNP	3.70	0.96 (2.01)	0.88 (0.57–1.58)	4.08	0.95 (2.01)	0.88 (0.58–1.51)	0.982
Di-2-ethylhexyl phthalate (DEHP)	MEHP	79.3	3.64 (4.87)	4.97 (1.82–11.13)	79.6	3.75 (4.69)	5.06 (1.89–10.21)	0.990
	MEHHP	99.3	16.01 (2.43)	16.13 (10.09–26.54)	99.3	16.4 (2.25)	16.09 (10.63–25.75)	0.788
	MEOHP	98.2	13.54 (2.51)	14.14 (8.33–24.05)	98.6	13.26 (2.28)	13.69 (8.36–21.51)	0.653
	**ΣDEHP** ^**†**^	**NA**	**0.13 (2.37)**	**0.13 (0.08–0.21)**	**NA**	**0.13 (2.20)**	**0.12 (0.08–0.20)**	**0.921**
	MECPP^‡^	98.9	22.74 (2.86)	22.79 (15.15–38.12)	99.3	22.47 (2.21)	22.46 (15.84–33.19)	0.823
	MCMHP^‡^	97.0	5.58 (2.77)	5.83 (3.75–9.25)	97.3	5.08 (2.52)	5.51 (4.02–7.92)	0.372

^†^ ΣDEHP was estimated exposure of DEHP by summation of MEHP, MEHHP, and MEOHP and presented as *μ*mol/ g creatinine.

^‡^ Sample size was only 214 for the females and 222 for the males, respectively.

DR%, detection rate (% for values > limit of detection). GM, geometric mean. GSD, geometric standard deviation. NA, non-applicable.

Table 2 presents descriptive statistics of AGD and cord-blood sex steroid hormone levels. Among 564 newborns, 262 received AGD measures. Male newborns had significant higher cord-blood FSH and SHBG levels but similar progesterone, E2, and frTT levels than female newborns. Mothers with male babies had significantly higher serum progesterone but similar E2 and TT than mothers with female babies.

**Table 2:** Male and female newborn’s anogenital distances, cord-blood and maternal sex steroid hormone levels.

AGDs and sex steroid hormones	Male newborns (N = 294)				Female newborns (N = 270)				*P* -value
	n	DR %	GM (GSD)	Median (Q1-Q3)	n	DR %	GM (GSD)	Median (Q1-Q3)	
**Child’s information**									
- AGD (mm) ^a^	141		19.60 (6.39)	19.15 (16.20–22.75)	122		12.64 (4.36)	12.23 (10.10–15.00)	
- **Maternal hormones**									
Progesterone (ng/mL)	273	98.20	145.5±1.92	134.7 (101.5, 213.4)	244	98.79	129.0±1.77	127.0 (87.74, 181.6)	0.011
E2 (pg/mL)	269	96.76	14,472±1.99	14,675 (11,663, 19,647)	244	98.79	15,214±2.05	15,598 (12,836, 20,963)	0.095
TT (ng/dL)	277	99.64	90.02±1.65	93.00 (65.00, 124.0)	247	100.0	90.02±1.60	86.00 (63.00, 124.0)	0.592
- Cord-blood hormones									
Progesterone (ng/mL)	290	98.64	804.3 (1.86)	840.3 (614.9–1,141)	269	99.63	772.8 (1.68)	788.9 (582.5–1,072)	0.134
E2 (pg/mL)	289	98.30	6,568 (1.93)	7,088 (4,467–10,183)	269	99.63	5,884 (2.03)	6,250 (3,901–9,250)	0.076
FSH (mIU/mL)	291	89.69	0.90 (2.36)	1.00 (0.60–1.60)	268	56.72	0.36 (2.61)	0.35 (0.15–0.65)	<0.001
frTT (ng/dL)	232	78.91	4.22 (1.63)	4.29 (3.51–5.71)	205	75.93	4.18 (1.80)	4.26 (3.23–5.93)	0.954
SHBG (nmol/L)	293		38.47 (1.82)	36.4 (30.9–46.8)	268		33.74 (1.93)	34.2 (26.1–42.9)	<0.001

^a^ AGD was only measured in a subgroup of study participants (n = 263). AGD, anogenital distance. DR, detection rate. GM, geometric mean. GSD, geometric standard deviation. E2, estradiol. TT, total testosterone. FSH, follicle stimulating hormone. frTT, free-form testosterone. SHBG, sex hormone-binding globulin. Q1, 1^st^ quartile. Q3, 3^rd^ quartile.

**Table 3** shows the relationship between prenatal phthalate metabolites exposure and AGD in male and female newborns. In male newborns, median exposure levels of MMP, MBzP, MEOHP, and ΣDEHP were associated with 3.20 mm, 3.82 mm, 3.70 mm, and 4.04 mm shorter AGD compared with low exposure levels of these metabolites, respectively. Although the averaged AGDs in babies with high exposure levels of abovementioned phthalate metabolites were shorter than that in babies with low exposure levels, but the differences were not statistically significant. In female newborns, prenatal exposure levels of phthalate metabolites were not significantly associated with alterations of AGD. However, as we replaced the categorical phthalate metabolite levels with continuous concentrations in natural logarithm scale and reperformed the analyses, no phthalate metabolite was statistically significantly associated with AGDs in both male and female newborns (S3 Table).

**Table 3:** Associations of prenatal exposure of phthalate metabolites with AGD in male and female newborns.

Phthalate metabolites (μg/ g creatinine)	Level^a^	Male newborns (n = 124)			Female newborns (n = 113)		
		Adjusted β	(95% CI)	*P*	Adjusted β	(95% CI)	*P*
**MMP**	M	**-3.20**	**(-6.27, -0.13)**	**0.041**	0.89	(-1.03, 2.81)	0.361
	H	-1.73	(-4.72, 1.26)	0.253	-1.82	(-3.86, 0.22)	0.080
**MEP**	M	-1.64	(-4.41, 1.13)	0.243	-0.13	(-2.17, 1.92)	0.902
	H	-0.20	(-3.26, 2.86)	0.898	1.03	(-1.05, 3.12)	0.329
**MnBP**	M	1.06	(-1.89, 4.01)	0.479	1.51	(-0.57, 3.6)	0.153
	H	0.31	(-2.78, 3.40)	0.843	1.93	(-0.24, 4.1)	0.080
**MiBP**	M	0.23	(-2.71, 3.16)	0.878	1.61	(-0.51, 3.72)	0.135
	H	2.20	(-0.77, 5.16)	0.145	0.84	(-1.27, 2.95)	0.432
**MBzP**	M	**-3.82**	**(-6.75, -0.88)**	**0.011**	-0.37	(-2.36, 1.62)	0.716
	H	-1.27	(-3.96, 1.42)	0.351	0.54	(-1.53, 2.61)	0.605
**MEHP**	M	-2.18	(-5.14, 0.78)	0.147	-0.38	(-2.34, 1.58)	0.705
	H	-2.21	(-5.38, 0.96)	0.171	1.15	(-0.93, 3.24)	0.274
**MEHHP**	M	-2.13	(-4.87, 0.61)	0.126	-1.70	(-3.59, 0.19)	0.077
	H	-0.91	(-3.93, 2.10)	0.551	1.36	(-0.75, 3.47)	0.205
**MEOHP**	M	**-3.70**	**(-6.40, -0.99)**	**0.008**	-1.37	(-3.28, 0.53)	0.155
	H	-0.17	(-3.11, 2.77)	0.908	0.58	(-1.56, 2.73)	0.590
**ΣDEHP**	M	**-4.04**	**(-6.84, -1.24)**	**0.005**	-1.71	(-3.57, 0.16)	0.073
(μmol/ g creatinine)	H	-2.22	(-5.25, 0.81)	0.150	0.71	(-1.46, 2.87)	0.519

^a^ Low exposure level as the reference.

ΣDEHP was estimated exposure of DEHP by summation of MEHP, MEHHP, and MEOHP exposure level. M, median exposure level. H, highest exposure level. Adjusted β, regression coefficient adjusted for maternal age at enrollment and maternal education status, household income, maternal exercise habit, and pre-pregnancy BMI,. CI, confidence interval. *P*, p-value.

**Fig 2:**
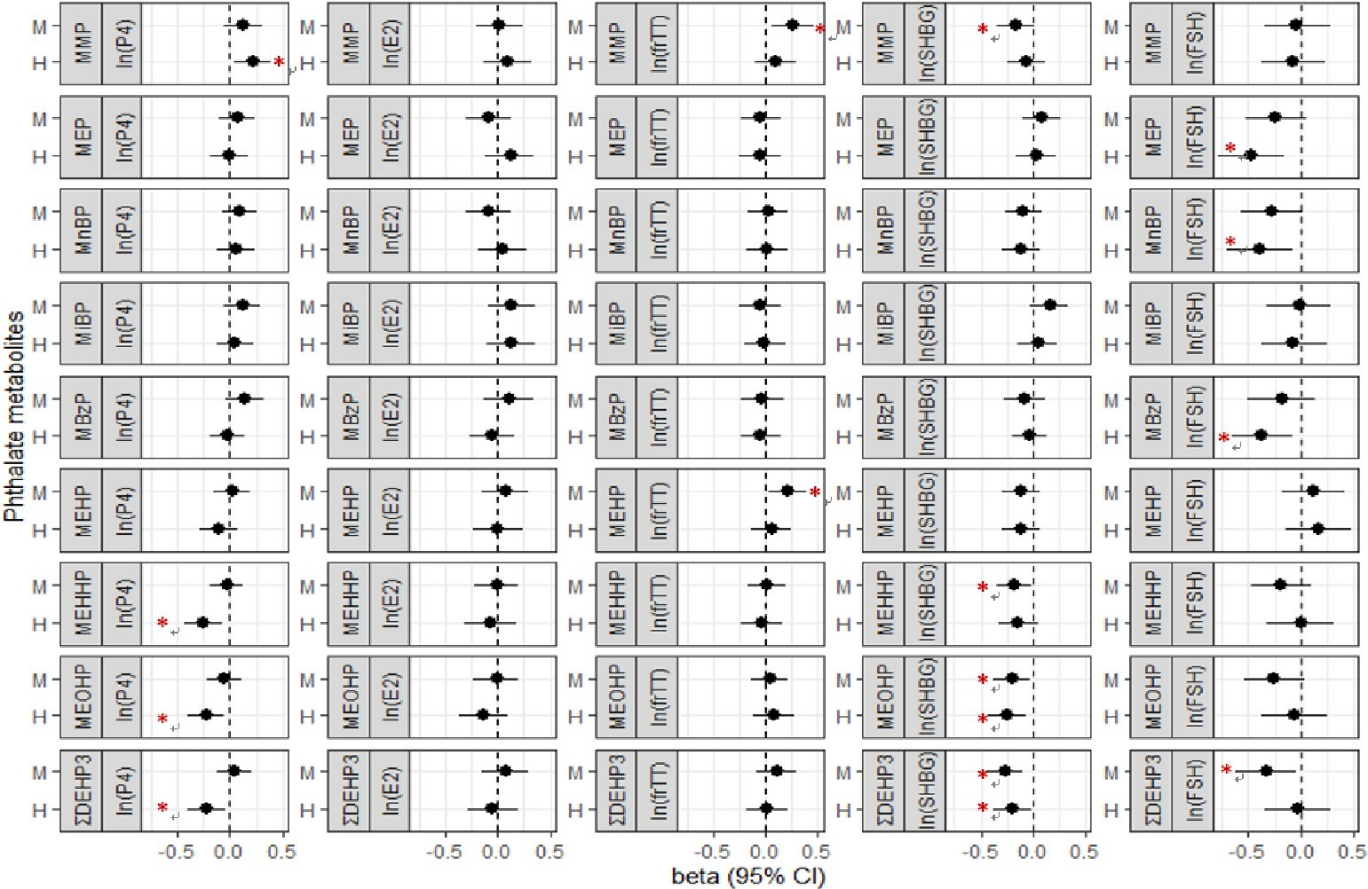
Adjusted changes of male newborn’s sex steroid hormones in log scale corresponding to exposure levels of phthalate metabolites from low to median or high levels, respectively. ΣDEHP was estimated exposure of DEHP by summation of MEHP, MEHHP, and MEOHP exposure level. P4, progesterone. E2, estradiol. frTT, free-form testosterone. SHBG, sex hormone-binding globulin. FSH, follicle stimulating hormone. M, median exposure level. H, highest exposure level. CI, confidence interval. Beta, regression coefficient adjusted for maternal age at enrollment and maternal education status, household income, maternal exercise habit, and pre-pregnancy BMI.

**Fig 2** shows the adjusted changes of cord-blood sex steroid hormones in male newborns, which were corresponding to median and high prenatal exposure levels compared to low exposure levels of phthalate metabolites. Higher exposure of MMP was associated with increased level of progesterone, frTT, and decreased level of SHBG. Higher exposure of MEP, MnBP, and MBzP were associated with decreased level of FSH. Higher exposure of MEHP, MEHHP, and MEOHP were associated with decreased levels of progesterone and SHBG, though only higher MEHP levels was associated with increased levels of frTT. Increased exposure of ΣDEHP was associated with decreased levels of progesterone, SHBG, and FSH.

**Fig 3:**
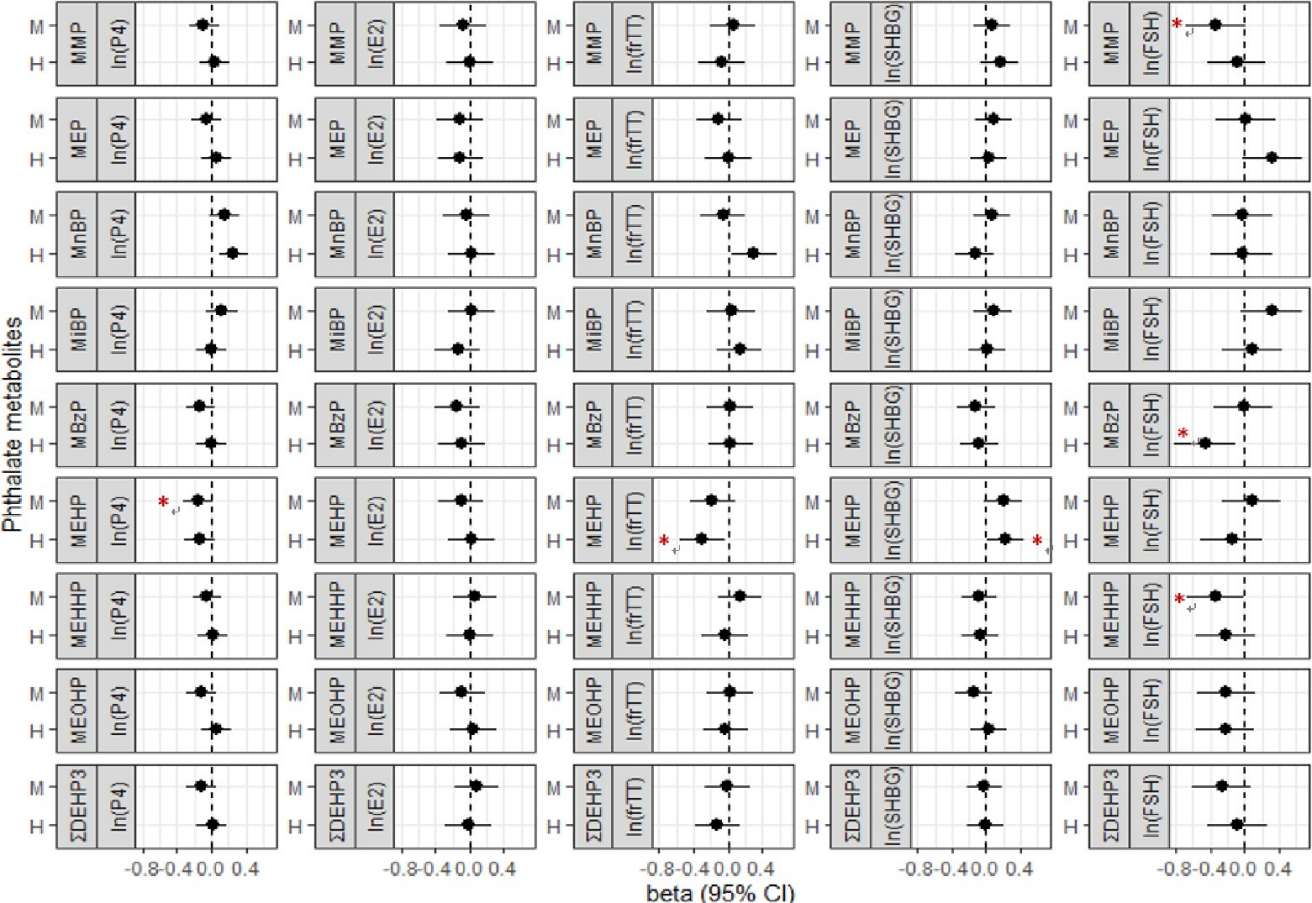
Adjusted changes of female newborn’s sex steroid hormones in log scale corresponding to exposure levels of phthalate metabolites from low to median or high levels, respectively. ΣDEHP was estimated exposure of DEHP by summation of MEHP, MEHHP, and MEOHP exposure level. P4, progesterone. E2, estradiol. frTT, free-form testosterone. SHBG, sex hormone-binding globulin. FSH, follicle stimulating hormone. M, median exposure level. H, highest exposure level. CI, confidence interval. Beta, regression coefficient adjusted for maternal age at enrollment and maternal education status.

Similarly, **Fig 3** presents adjusted changes of cord-blood sex steroid hormones in female newborns. Higher exposure of MnBP was associated with increased level of progesterone and frTT. Higher exposures of MMP, MBzP, and MEHHP were associated with decreased level of FSH. Higher exposure to MEHP was associated with decreased level of progesterone and frTT but increased level of SHBG. However, as we replaced the categorical phthalate metabolite levels with continuous concentrations in natural logarithm scale and reperformed the analyses, some of the significant associations between phthalate metabolite levels and cord-blood sex steroid hormone levels became statistically insignificant in male and female newborns (S4 Table).

To exploring the potential mediation of cord-blood sex steroid hormones, we also examined the relationships between cord-blood and maternal sex steroid hormone and AGD in **S5 Table.** Almost all sex hormones tended to be inversely associated with AGD in male and female newborns respectively, but none of them achieved statistically significance.

## Discussions

Our study results confirmed the associations between maternal urinary phthalate metabolites and alterations in AGD in male newborns and reported no significant association in female newborns. We also found interference of maternal phthalate exposure on cord-blood sex steroid hormones. Despite interference of phthalates on sex hormones had been widely reported among children, adolescents, and adults, studies examining the effect of maternal phthalate exposure on fetus hormones are quite limited [16]. However, we failed to link cord-blood and maternal sex hormone levels to the alterations of AGDs in our study. What other factors might mediate the effect of prenatal exposures to phthalates on newborn’s AGD needs further investigations.

### Alterations of AGD associated with prenatal phthalate exposures

Decreased AGD is a sensitive marker detecting anti-androgen effect [14]. Our study reported that median levels of maternal MMP, MBzP, MEOHP, and ΣDEHP were associated with shortened AGD in male newborns, which was aligned with findings in past literatures. Previous studies have reported that a shorter AGD was related to prenatal DEHP and DMP exposure [10, 11, 17] and anopenile distance was negatively associated with in-utero MEP, MiBP, and MBP [21] in male newborns. The underlying mechanism has been related to phthalates’ anti-androgen effect such as testosterone inhibition in the animal studies. Male litters of rat dams gavaged with high doses of DEHP or butyl benzyl phthalate were more likely to have shorter AGD and reduced testis weights [22, 23], suppressed fetal testosterone [23], and malformation of reproductive systems [15, 22]. However, evidence gap exists in the female newborns due to limited amounts of studies reported in females [16]. Our study findings were aligned to the only available epidemiologic studies in the past, neither of which found a significant effect of maternal exposure of phthalates on the females’ AGD at birth [11, 24–26]. However, we observed borderline significant increased AGD and borderline significant decreased AGD in female babies with median maternal MiBP and MEHHP, respectively. There was also a meta-analysis showing an increased female’s AGD in female babies with higher maternal MBzP levels [21]. Although the effect of maternal phthalate exposure on female’s AGD at birth seems not to be so pronounced as that in males, more studies with sufficient sample size for baby girls are expected to further confirm the effect in females.

### Associations between maternal urinary phthalate metabolites and cord-blood sex steroid hormones

Evidences have widely suggested that the reproductive toxicity of phthalates involves dysfunction of hypothalamic-pituitary-gonadal (HPG) axis [27]. Although the actual mechanism is still unclear, the reproductive toxicity of phthalates may be associated with its anti-androgenic property and seems to be more frequently reported in males than in females [17, 28].The upstream changes in HPG are associated with the abnormal expressions of peroxisome proliferator-activated receptors, aryl hydrocarbon receptors, and insulin receptors, which may alter the release of gonadal-releasing hormones and gonadotropins and induce cell damages [29], subsequently disrupt synthesis of sex steroid hormones and functions or morphologic changes of reproductive organ. The reproductive disrupting effect of phthalates can be even transgenerational to the unexposed offspring in animal studies, but it still needs further confirmed in humans [27, 30].

To the best of our knowledge, there were only two previous studies investigating the effect of maternal phthalate exposures on cord-blood sex steroid hormones [12, 13] and a cross-sectional study which measured phthalate metabolites and fetal hormone levels in amniotic fluid [31]. Among the two studies measured hormone levels in cord-blood, one study measured phthalate metabolites in maternal serum [12], another study was the only one measured phthalate metabolites in maternal urine which is now considered as the most appropriate matrix for phthalate exposure assessment [13]. Lin and colleagues analyzed the data of a birth cohort study conducted in 2001 in Taiwan, it was about ten years before the 2011 phthalate-tainted foodstuff episode uncovered in Taiwan. Our study enrolled study participants in 2012, at the time the control measures had been intervened. After that time, phthalate exposures in pregnant women have been reduced. The median levels of MEHP and DEHP in our study cohort (MEHP 5.0 μg/g creatinine, DEHP 0.13 μmol/g creatinine) was lower than that in Lin’s study (19.1 μg/g creatinine, DEHP 0.14 μmol/g creatinine, measured in 2000–2001) [13] and Kuo’s study (11.9 μg/g creatinine, DEHP 0.19 μmol/g creatinine, measured in 2009) in Taiwan [32] **(S3 Fig)**.

Although maternal phthalate metabolites levels in our study were lower than that in previous studies in Taiwan, most phthalate metabolites were among the levels reported from Japan and US **(S3 Fig)**. Under the exposure levels reported in our study, we found that maternal DEHP metabolites were in an inverse relationship with cord-blood progesterone and FSH in male and female newborns. FSH stimulate Sertoli cells proliferation and sperms production with testosterone (TT) in prepuberty [33]. Our findings were consistent with experimental study that showed reduced Sertoli cell number and inhibited cell proliferations in rodents gavaged with DEHP [34]. It was also partially aligned with Araki’ study findings in HCS. Araki et al. found an inverse association of serum MEHP with cord-blood progesterone and inhibin B (an FSH-regulated functional marker secreted by Sertoli cells in testes), but not with FSH levels, in male newborns [12]. Their findings may suggest a functional reduction of Sertoli cells associated with maternal DEHP levels in serum. Jensen et al. only examined effects of MECPP in male newborns since it was the most abundant metabolite of DEHP detected in amniotic fluid. In contrary to our findings, their study observed an increased progesterone associated with higher MECPP [31]. However, the study determined the sex hormones levels in amniotic fluid samples; their observations may not be directly compared with our study results. The anti-androgen effect of DEHP has been widely observed in cross-sectional study in adults [35, 36]. However, our study also observed that elevated maternal DEHP metabolites was associated with higher cord-blood frTT and lower SHBG in male newborns as well as lower frTT and higher SHBG in female newborns. The tendency toward decreased frTT of maternal phthalate exposures in female newborns has also been observed in Lin’s study, which showed an inverse association between maternal DEHP exposure and cord-blood frTT and frTT/E2 in female newborns, but they did not find that association in male newborns [13]. Jensen’ study findings in male fetuses were similar to ours, showing that amniotic fluid MECPP levels was positively associated with TT levels [31]. Araki’s study did not find any significant effect of maternal serum MEHP on cord-blood TT, SHBG and TT/SHBG levels both in males and females [12]. Similar to DEHP, maternal MMP was also related to higher cord-blood frTT and lower SHBG in male newborns but not in female newborns in our study. Araki’s study and Jensen’s study did not report the effect of MMP. The limited previous studies showed that maternal MMP was not associated with frTT, TT, or frTT/E2 levels in cord-blood [13] and in 2-, 5-, 8-, and 11-year-old children’s serum [37]. In terms of cord-blood frTT and SHBG, our study seemed to suggest an androgenic effect in males and anti-androgenic effect in females of maternal DEHP and MMP, in contrary to the effect in adults or cross-sectional study. The underlying mechanism of our study results is unclear and needs further study to confirm.

Our study revealed that cord-blood FSH levels was the most affected sex hormone by maternal phthalate exposures. We particularly noted that both male and female’s cord-blood FSH levels decreased as maternal MBzP increased. Similar results have been found in a study assessing sex hormones levels in minipuberty [38]. FSH stimulates Sertoli cells proliferation and promotes sperm production with TT in prepuberty [33]. In male adults, MBzP was reported to be negatively associated with lower sperm concentration [39] and lower sperm motility [17]. Our study findings suggest that prenatal MBzP exposure may inhibit early reproductive tract development in male and female newborns.

Pregnant women were suggested to avoid consume the packaged food with unsafe plastic wrap or containers [2, 40], and reduce the use of PCPs, i.e., body lotion, perfume, lip stick… etc., particularly the leave-on items [41, 42] and the products with long lasting fragrance. The careful consumption of packaged food and selective use of PCPs may protect themselves and their babies to minimize exposure levels of phthalates.

### Strengths and limitations

Our study has several strengths. First, this study was one of the few cohort studies investigating the hormone-disrupting effect of prenatal phthalate exposure on cord-blood sex hormones and the associations between cord-blood sex hormones and AGD in newborns. Second, our study provided an opportunity to continually monitor the impact of relatively low prenatal phthalate exposure on fetal development after the 2011 phthalate-tainted-foodstuff episode in Taiwan. Third, we examined the babies’ cord-blood sex hormone concentrations, avoiding confounding effect of postnatal exposures of hormone-disrupting chemicals in environment.

Our study has certain limitations. First, we only collected one-spot urine samples in the third trimester, which may not fully represent the average phthalate-exposure level during pregnancy or the effective exposure levels on alteration of AGD at birth. A subset of participants (n = 142) in our study who provided urine samples in each trimester of pregnancy showed a weak-to-moderate consistency of DEHP metabolites between the third and first trimesters (Spearman’s correlation coefficient [ρ] 0.23 [MEHP]– 0.28 [MEMHP]) and between the third and second trimesters (ρ 0.17 [MEHP]– 0.42 [MEHHP]), indicating the likelihood of phthalate-exposure misclassifications in our study; nevertheless, the misclassifications should be non-differential and biased observed associations toward the null. Second, our study sample size may not be sufficient and thus reduced the statistical power in some analyses. Third, as we replaced the categorical form of phthalate metabolite levels with continuous concentrations, the significant associations between prenatal phthalate metabolite levels and male newborn’s AGD became statistically insignificant. We believed that might be partly resulted from limited sample size in our study. Another possible reason might be that the potential non-linear non-monotonic effects of phthalates on AGD were obscured in linear models. However, whether the relationships between prenatal phthalate exposures in such relative low levels were non-linearly associated with newborn’s AGD should be further validated.

## Conclusions

Our study observed that maternal urinary levels of MMP, MBzP, and DEHP metabolites at the 3^rd^ trimester were associated with shortened AGD in male newborns. However, neither cord-blood or maternal sex steroid hormones significantly associated with newborn’s AGD. We also found the hormone-disrupting effect of prenatal phthalate exposure on cord-blood sex hormones, particularly interference of MMP, MnBP, MBzP and DEHP-metabolites on FSH should be of concern. Women aspiring for pregnancy should be alerted to the need for reduction of phthalate exposures, such as contaminated food [2, 40], spray paints, and leave-on PCP [41, 42].

## Supporting information

S1 Fig Directed acyclic graph (DAG) showing study framework and adjusted variables to elucidate causal relationship of prenatal phthalate exposure with newborns’ sex hormones (a) and newborns’ AGDs (b).
(TIF)

S2 Fig Maternal concentrations of phthalate metabolites between mothers who initially provided urinary samples at the 3rd trimester (n = 1204) and those who included in data analyses (n = 564 and n = 263). (TIF)

S3 Fig The median maternal concentrations of phthalate metabolites in pregnant women in our study and in previous literatures. (TIF)

S1 Table Project title and approval numbers from Institutional Review Boards in National Health Research Institute and 9 collaborated hospitals. (DOCX)

S2 Table Maternal sociodemographic characteristics and birth outcomes in association with in-utero exposure of phthalate metabolites. (DOCX)

S3 Table Associations of prenatal phthalate metabolite levels in natural log scale with AGDs in male and female newborns. (DOCX)

S4 Table Associations of maternal phthalate metabolites levels with cord-blood sex steroid hormone levels in male and female newborns. (DOCX)

S5 Table Associations of products of cord-blood sex hormones and maternal sex hormone concentrations with AGD in male and female newborns. (DOCX)

S1 Graphical abstract (TIF)
