# Supplementary figures and images for: Prenatal phthalate exposure and sex steroid hormones in newborns: Taiwan Maternal and Infant Cohort Study
Source: PLoS One. 2024 Mar 14;19(3):e0297631. doi: 10.1371/journal.pone.0297631 (PMC10939196; doi:10.1371/journal.pone.0297631)

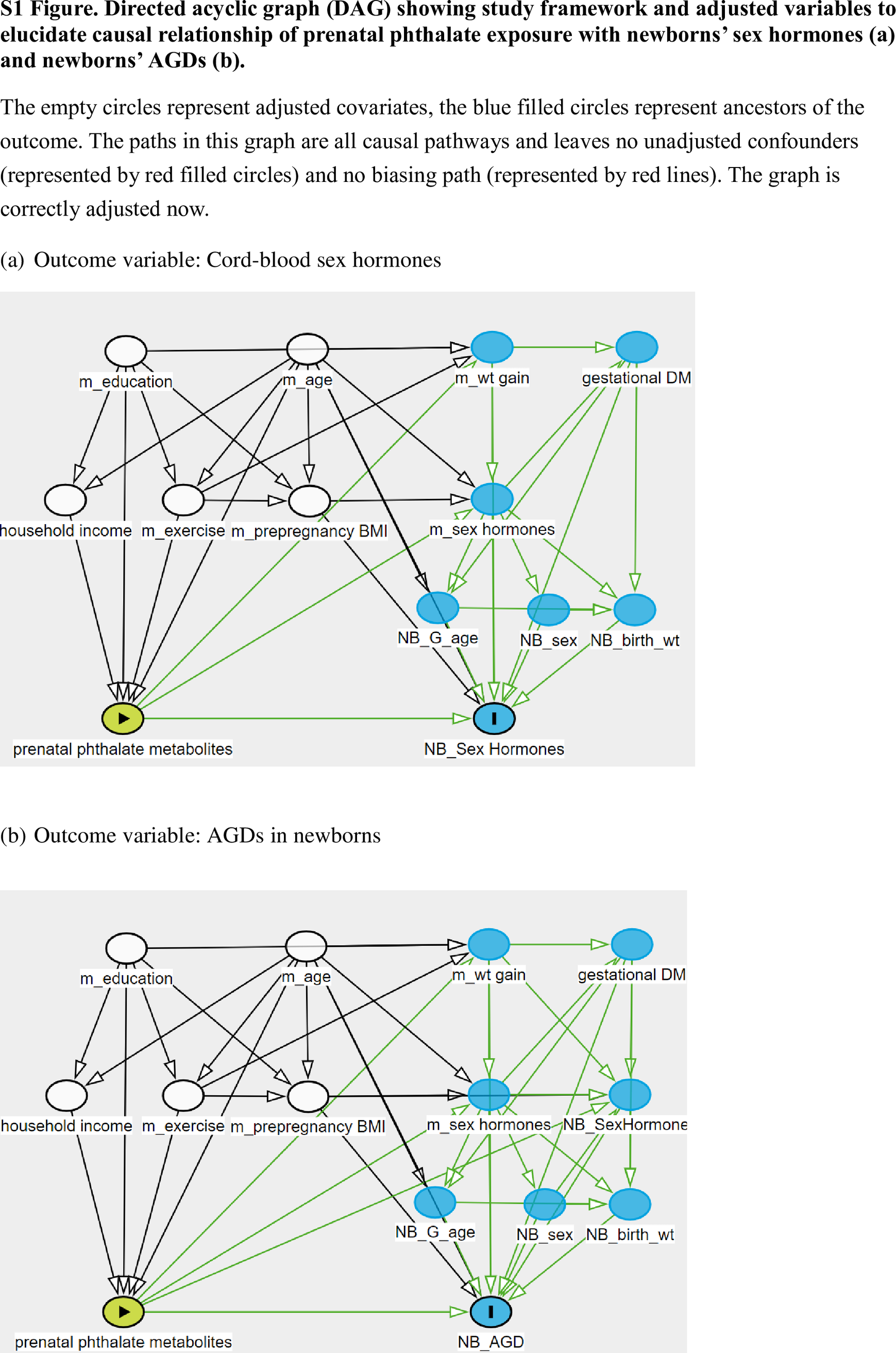

Supplement: S1 Fig — Directed acyclic graph (DAG) showing study framework and adjusted variables to elucidate causal relationship of prenatal phthalate exposure with newborns’ sex hormones (a) and newborns’ AGDs (b). (TIF) [file pone.0297631.s001.tif]

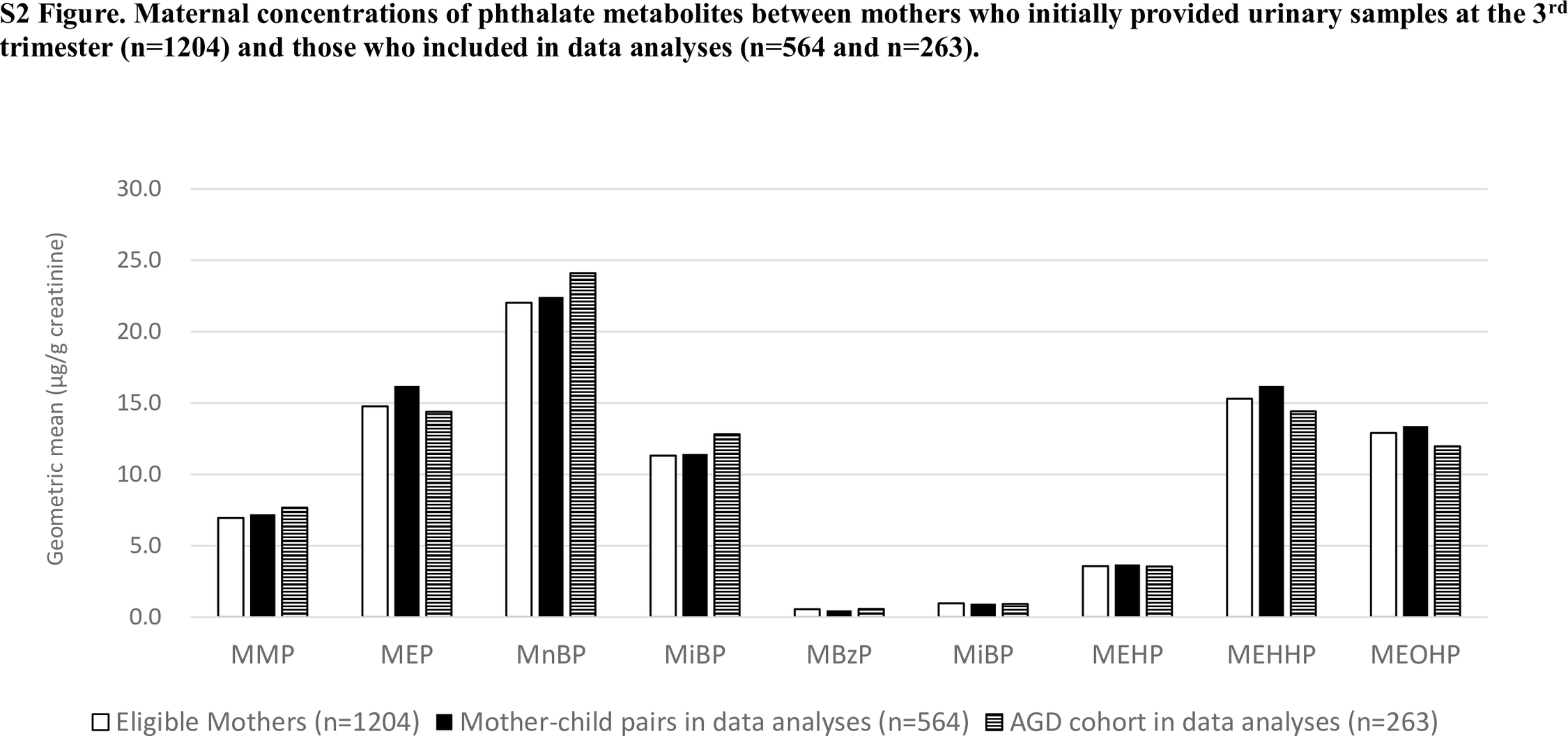

Supplement: S2 Fig — (TIF) [file pone.0297631.s002.tif]

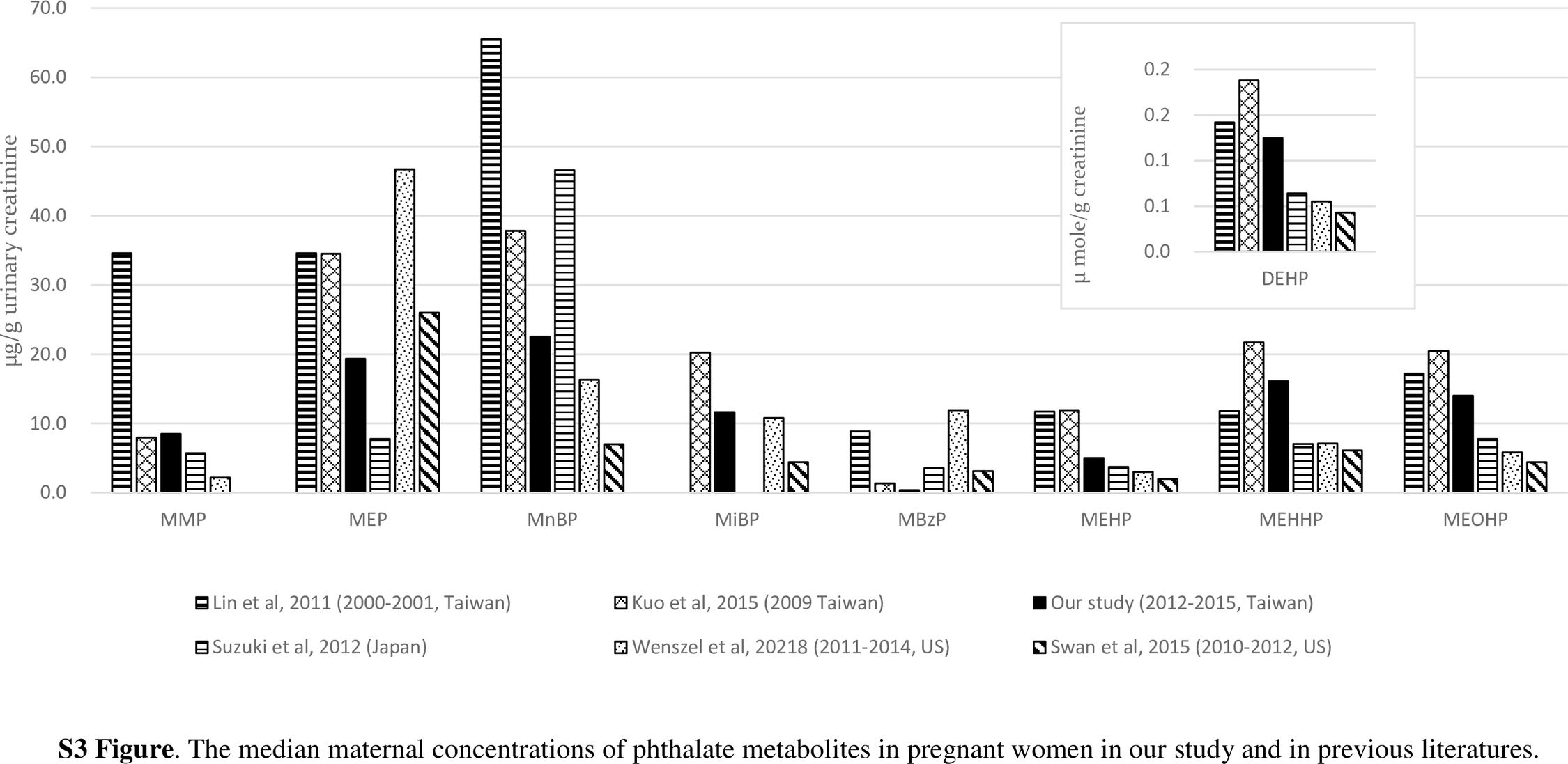

Supplement: S3 Fig — (TIF) [file pone.0297631.s003.tif]

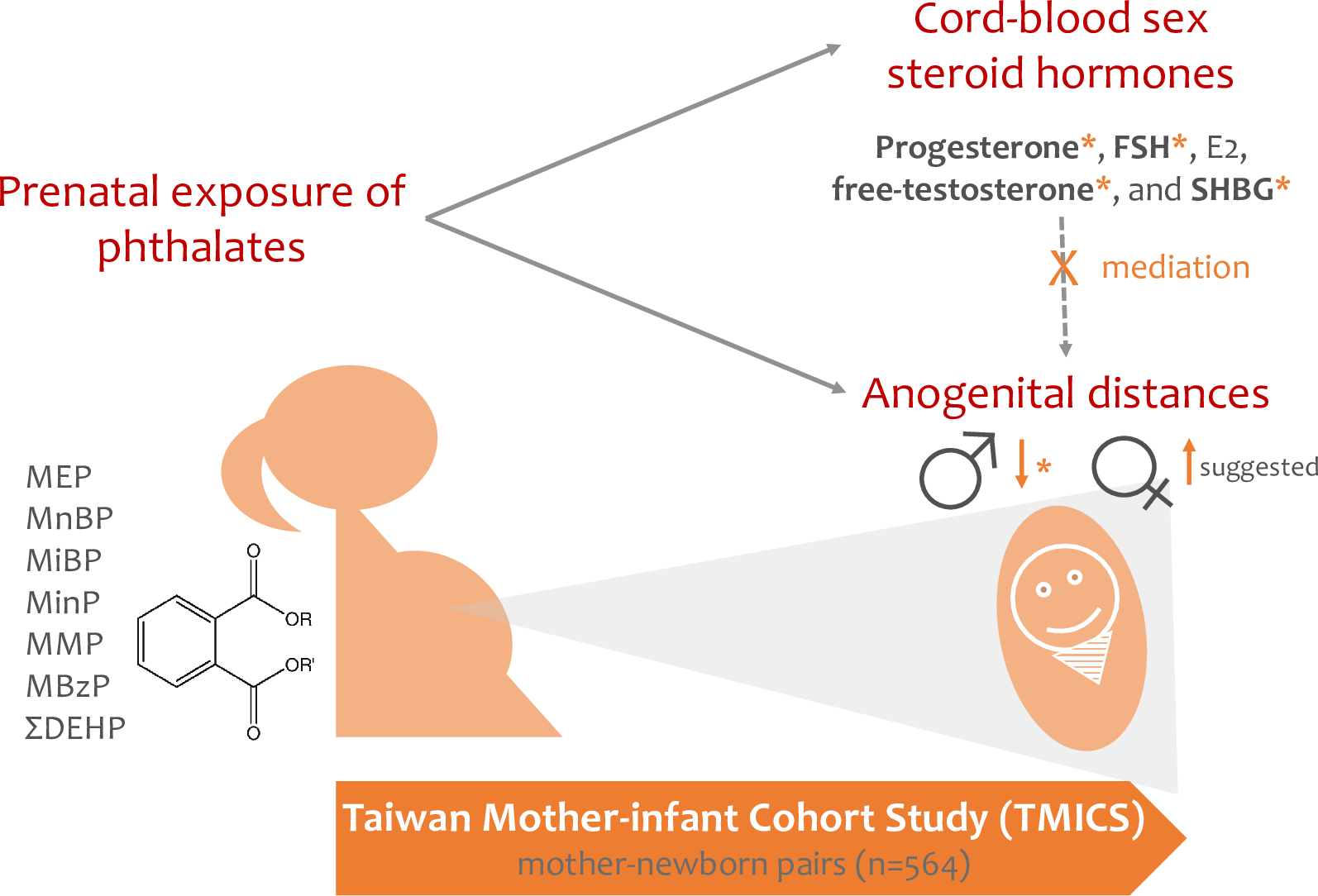

Supplement: S1 Graphical abstract — (TIF) [file pone.0297631.s009.tif]
