# Supplementary material for: Prenatal phthalate exposure and sex steroid hormones in newborns: Taiwan Maternal and Infant Cohort Study
Source: PLoS One. 2024 Mar 14;19(3):e0297631. doi: 10.1371/journal.pone.0297631 (PMC10939196; doi:10.1371/journal.pone.0297631)
Supplement: S1 Table — (DOCX) [file pone.0297631.s004.docx]

**S1 Table. Project title and approval numbers from Institutional Review Boards in National Health Research Institute and 9 collaborated hospitals.**

| **Research areas** | **Hospitals** | **IRB approval number** | **Title** |
| --- | --- | --- | --- |
| Northern area | Taipei Veterans General Hospital | 2012-08-004AY | A follow-up study of environmental toxins such as phthalate and its health effects on reproductive hormones, cognitive and developmental functions |
|  | Cathay General Hospital | CGH-P101049 | A follow-up study of environmental toxins such as phthalate and its health effects on reproductive hormones, cognitive and developmental functions |
|  | Taipei City Hospital | TCHIRB-1030332 | Protection and control of pollutant related effects for healthy growth, development and reproduction in the susceptible population of women and their children |
| Middle area | National Health Research Institute | EC1010501 | A follow-up study of environmental toxins such as phthalate and its health effects on reproductive hormones, cognitive and developmental functions |
|  | Hsinchu Cathay General Hospital | CGH-P101049  (same as that from Cathay General Hospital) | A follow-up study of environmental toxins such as phthalate and its health effects on reproductive hormones, cognitive and developmental functions |
|  | Chung Shan Medical University Hospital | CS12082 | A follow-up study of environmental toxins such as phthalate and its health effects on reproductive hormones, cognitive and developmental functions |
|  | Changhua Christian Hospital | 120618 | A follow-up study of environmental toxins such as phthalate and its health effects on reproductive hormones, cognitive and developmental functions |
| Southern area | E-DA Hospital | EMRP35101N | A follow-up study of environmental toxins such as phthalate and its health effects on reproductive hormones, cognitive and developmental functions |
|  | Kaohsiung Municipal Siaogan Hospital | KMUHIRB- 2012-11-02(I) | A follow-up study of environmental toxins such as phthalate and its health effects on reproductive hormones, cognitive and developmental functions |
| Eastern area | Hualien Tzu Chi Hospital | ACT-IRB101-11 | A follow-up study of environmental toxins such as phthalate and its health effects on reproductive hormones, cognitive and developmental functions |

IRB, Institutional Review Board
