# Supplementary material for: Prenatal phthalate exposure and sex steroid hormones in newborns: Taiwan Maternal and Infant Cohort Study
Source: PLoS One. 2024 Mar 14;19(3):e0297631. doi: 10.1371/journal.pone.0297631 (PMC10939196; doi:10.1371/journal.pone.0297631)
Supplement: S2 Table — (DOCX) [file pone.0297631.s005.docx]

**S2 Table. The associations of maternal sociodemographic characteristics and birth outcomes with prenatal exposure of phthalate metabolites.**

| Characteristics |  | N | MMP | MEP | MnBP | MiBP | MBzP |
| --- | --- | --- | --- | --- | --- | --- | --- |
|  |  |  | Median (IQR) | Median (IQR) | Median (IQR) | Median (IQR) | Median (IQR) |
| Maternal characteristics |  |  |  |  |  |  |  |
| Age at enrollment (yrs) | <25 | 36 | 8.94 (5.42–17.09) | **26.28 (13.8–47.56)*** | 31.72 (13.46–44.64) | 12.3 (8.82–26.33) | 0.69 (0.11–1.77) |
|  | 25-34 | 413 | 8.37 (4.83–15.2) | **21.11 (8.55–65.75)** | 22.53 (13.53–34.49) | 11.43 (6.98–19.88) | 0.42 (0.12–1.94) |
|  | >=35 | 115 | 8.55 (3.53–14.15) | **13.35 (5.79–35.34)** | 20.54 (13.14–34.86) | 11.3 (6.87–18.31) | 0.24 (0.11–1.71) |
| Regular exercise | no | 269 | 7.83 (4.11–15.2) | 18.11 (7.75–53.42) | 23.21 (14.38–37.19) | 12.55 (7.9–22.55) | **0.80 (0.13–2.38)*** |
|  | yes | 162 | 9.57 (5.74–15.8) | 16.47 (7.77–55.48) | 22.72 (13.53–35.2) | 11.77 (7.58–18.65) | **0.23 (0.10–1.79)** |
| Education level | high school and below | 131 | 8.59 (4.48–14.92) | 25.64 (7.77–66) | **27.18 (13.53–45.41)**** | 12.85 (6.47–23.87) | 0.24 (0.10–1.59) |
|  | college and above | 430 | 8.38 (4.53–15.26) | 17.41 (7.77–51.12) | **21.69 (13.49–33.97)** | 11.04 (7.11–18.89) | 0.39 (0.12–1.99) |
| Annual household income (USD) | <16,666 | 94 | 7.77 (5.6–14.78) | 18.91 (7.07–54.08) | 27.15 (16.93–41.21) | 13.75 (8.32–24.72) | 0.46 (0.11–2.08) |
|  | 16,666 to <33,333 | 177 | 9.58 (4.61–17.63) | 15.46 (7.62–46.22) | 23.07 (14.08–32.79) | 11.8 (7.07–18.49) | 0.73 (0.12–2.03) |
|  | >=33,333 | 124 | 8.83 (3.56–14.26) | 21.24 (8.37–75.12) | 21.43 (13.75–34.65) | 12.65 (8.01–20.95) | 0.43 (0.14–2.36) |
| Maternal weight gain (kg) | <8 | 192 | 8.82 (4.6–15.69) | 18.39 (7.43–53) | **24.68 (14.76–42.27)*** | 11.74 (7.66–18.76) | 0.54 (0.13–2.01) |
|  | 8 to < 12 | 202 | 7.38 (4.32–14.25) | 19.27 (8.12–53.42) | **21.04 (13.07–33.58)** | 12.57 (7.18–22.56) | 0.27 (0.11–1.77) |
|  | >=12 | 154 | 9.55 (4.61–15.82) | 21.93 (7.77–59) | **21.64 (12.62–31.3)** | 10.02 (6.52–16.11) | 0.32 (0.11–1.72) |
| Child characteristics |  |  |  |  |  |  |  |
| Child sex | female | 270 | 7.79 (4.14–14.26) | 19.22 (7.84–55.48) | 22.53 (13.58–35.82) | 11.77 (6.85–21.09) | 0.34 (0.11–1.71) |
|  | male | 294 | 8.95 (4.89–15.79) | 19.41 (7.75–54.77) | 22.31 (13.26–35.7) | 11.29 (7.26–18.65) | 0.38 (0.11–2.14) |
| Birth weight (g) | <2500 | 24 | 8.05 (3.58–15.34) | 12.77 (2.67–52) | 23.18 (13.15–48.15) | 14.59 (7.15–24.77) | 0.40 (0.14–1.86) |
|  | 2500 to <3200 | 297 | 8.98 (5.12–14.83) | 19.92 (8.89–52.49) | 22.53 (13.83–35.36) | 11.41 (7.07–18.46) | 0.32 (0.11–1.86) |
|  | >=3200 | 221 | 8.23 (4.32–15.26) | 19.37 (7.65–59) | 22.7 (14.08–36.54) | 11.84 (7.1–21.87) | 0.46 (0.12–1.94) |
| Gestational age (weeks) | <38 | 29 | 7.45 (4.07–15.06) | 29.06 (6.73–54.08) | 18.74 (12–28.61) | 9.93 (5.47–15.39) | 0.52 (0.13–1.78) |
|  | >=38 | 513 | 8.68 (4.61–15.2) | 18.89 (7.77–53.51) | 22.79 (13.98–35.82) | 11.88 (7.19–20.02) | 0.33 (0.11–1.93) |

**S2 Table (continued).**

| Characteristics |  | N | MEHP | MEHHP | MEOHP | ΣDEHP3 |
| --- | --- | --- | --- | --- | --- | --- |
|  |  |  | Median (IQR) | Median (IQR) | Median (IQR) | Median (IQR) |
| Maternal characteristics |  |  |  |  |  |  |
| Age at enrollment (yrs) | <25 | 36 | 4.00 (1.22–9.56) | 18.45 (10.45–29.28) | 17.34 (8.64–26.18) | 0.13 (0.08–0.21) |
|  | 25-34 | 413 | 5.12 (1.89–10.34) | 15.53 (10.09–25.54) | 13.43 (8.21–20.89) | 0.12 (0.08–0.2) |
|  | >=35 | 115 | 5.01 (1.79–12.15) | 16.91 (10.42–27.53) | 14.39 (8.45–23.7) | 0.13 (0.08–0.21) |
| Regular exercise | no | 269 | 5.32 (2.29–10.25) | 13.87 (9.59–24.69) | 11.81 (7.54–20.66) | 0.11 (0.07–0.19) |
|  | yes | 162 | 3.96 (0.71–9.14) | 13.74 (8.24–21.11) | 12.49 (7.74–19.21) | 0.11 (0.06–0.17) |
| Education level | high school and below | 131 | 5.21 (2.01–12.21) | **20.21 (11.4–31.16)**** | **16.85 (10.59–25.02)**** | **0.14 (0.09–0.23)**** |
|  | college and above | 430 | 5.01 (1.59–10.2) | **14.53 (9.94–24.7)** | **13.17 (8.01–20.8)** | **0.11 (0.08–0.18)** |
| Annual household income (USD) | <500 | 94 | 5.64 (2.11–9.66) | **14.74 (10.29–24.9)*** | **13.87 (9.1–22.23)*** | **0.12 (0.08–0.19)*** |
|  | 500 to <1000 | 177 | 5.44 (1.43–10.95) | **15.56 (10.42–24.18)** | **13.11 (8.19–19.77)** | **0.12 (0.08–0.19)** |
|  | >=1000 | 124 | 4.96 (1.87–9.5) | **12.07 (8.17–20.17)** | **9.95 (6.49–17.32)** | **0.10 (0.06–0.15)** |
| Maternal weight gain (kg) | <8 | 192 | 5.02 (2.17–10.49) | 17.94 (11.25–28.78) | 14.43 (9.42–24.94) | 0.13 (0.09–0.23) |
|  | 8 to < 12 | 202 | 4.82 (1.74–10.7) | 14.46 (9.7–25.02) | 12.73 (7.79–20.8) | 0.11 (0.08–0.18) |
|  | >=12 | 154 | 5.14 (1.33–9.81) | 17.04 (10.42–25.54) | 14.99 (8.39–21.46) | 0.13 (0.08–0.2) |
| Child characteristics |  |  |  |  |  |  |
| Child sex | female | 270 | 4.96 (1.82–11.13) | 16.13 (10.09–26.54) | 14.14 (8.33–24.05) | 0.13 (0.08–0.21) |
|  | male | 294 | 5.06 (1.89–10.21) | 16.09 (10.63–25.75) | 13.69 (8.35–21.51) | 0.12 (0.08–0.19) |
| Birth weight (g) | <2500 | 24 | 2.98 (0.6–6.75) | 12.39 (8.19–27.02) | 11.76 (7.74–24.97) | 0.10 (0.06–0.19) |
|  | 2500 to <3200 | 297 | 5.06 (1.87–11.31) | 17.06 (11.17–27.53) | 14.24 (9.27–23.1) | 0.13 (0.08–0.21) |
|  | >=3200 | 221 | 5.21 (2.62–9.53) | 15.73 (9.92–25.75) | 14.40 (8.07–21.76) | 0.12 (0.08–0.2) |
| Gestational age (weeks) | <38 | **29** | **3.02 (1.04–5.26)*** | 13.67 (9.1–27.09) | 11.19 (7.67–21.35) | 0.11 (0.07–0.19) |
|  | >=38 | **513** | **5.25 (1.94–10.88)** | 16.55 (10.7–26.54) | 14.24 (8.5–22.99) | 0.13 (0.08–0.21) |

IQR, interquartile range. NTD, New Taiwan Dollars. * 0.01<=p<0.05, ** 0.001<p<0.01, ***p<0.001
