# Supplementary material for: Prenatal phthalate exposure and sex steroid hormones in newborns: Taiwan Maternal and Infant Cohort Study
Source: PLoS One. 2024 Mar 14;19(3):e0297631. doi: 10.1371/journal.pone.0297631 (PMC10939196; doi:10.1371/journal.pone.0297631)
Supplement: S3 Table — (DOCX) [file pone.0297631.s006.docx]

**S3 Table.** **Associations of prenatal phthalate metabolite levels in natural log scale with AGDs in male and female newborns.**

| Phthalate metabolites  (μg/ g creatinine) | Male newborns (n=124) | | |  | Female newborns (n=113) | | |
| --- | --- | --- | --- | --- | --- | --- | --- |
|  | Adjusted β | (95% CI) | *P* |  | Adjusted β | (95% CI) | *P* |
| ln_MMP | -0.58 | (-1.44, 0.29) | 0.193 |  | -0.33 | (-0.93, 0.28) | 0.288 |
| ln_MEP | 0.02 | (-0.61, 0.65) | 0.944 |  | 0.01 | (-0.41, 0.43) | 0.975 |
| ln_MnBP | -0.38 | (-1.60, 0.84) | 0.537 |  | 0.39 | (-0.7, 1.48) | 0.479 |
| ln_MiBP | 0.35 | (-1.12, 1.82) | 0.638 |  | -0.02 | (-0.95, 0.92) | 0.975 |
| ln_MBzP | -0.41 | (-1.07, 0.26) | 0.226 |  | -0.02 | (-0.51, 0.47) | 0.940 |
| ln_MEHP | -0.15 | (-0.95, 0.64) | 0.701 |  | 0.18 | (-0.33, 0.68) | 0.486 |
| ln_MEHHP | -0.58 | (-2.17, 1.01) | 0.469 |  | 0.26 | (-0.73, 1.24) | 0.607 |
| ln_MEOHP | -0.55 | (-1.93, 0.84) | 0.436 |  | 0.30 | (-0.65, 1.25) | 0.530 |
| ln_ΣDEHP  (μmol/ g creatinine) | -0.52 | (-2.05, 1.00) | 0.499 |  | 0.33 | (-0.66, 1.32) | 0.510 |

ΣDEHP was estimated exposure of DEHP by summation of MEHP, MEHHP, and MEOHP exposure level. Adjusted β, regression coefficient adjusted for maternal age at enrollment, maternal education status, household income, maternal exercise habit, and pre-pregnancy BMI. CI, confidence interval. P, p-value.
