# Supplementary material for: Prenatal phthalate exposure and sex steroid hormones in newborns: Taiwan Maternal and Infant Cohort Study
Source: PLoS One. 2024 Mar 14;19(3):e0297631. doi: 10.1371/journal.pone.0297631 (PMC10939196; doi:10.1371/journal.pone.0297631)
Supplement: S4 Table — (DOCX) [file pone.0297631.s007.docx]

**S4 Table. Associations of maternal phthalate metabolites levels with cord-blood sex steroid hormone levels in male and female newborns.**

| **Newborn's**  **sex** | **Phthalate metabolites**  **(μg/ g creatinine)** | **ln(P4)** | | |  | **ln(E2)** | | |  | **ln(frTT)** | | |  | **ln(SHBG)** | | |  | **ln(FSH)** | | |
| --- | --- | --- | --- | --- | --- | --- | --- | --- | --- | --- | --- | --- | --- | --- | --- | --- | --- | --- | --- | --- |
|  |  | Adj-*β* | 95% CI | *P* |  | Adj-*β* | 95% CI | *P* |  | Adj-*β* | 95% CI | *P* |  | Adj-*β* | 95% CI | *P* |  | Adj-*β* | 95% CI | *P* |
| Male | **ln_MMP** | **0.06** | **(0.00, 0.12)** | **0.045** |  | 0.00 | (-0.07, 0.08) | 0.898 |  | 0.05 | (-0.03, 0.12) | 0.233 |  | -0.02 | (-0.08, 0.04) | 0.444 |  | -0.04 | (-0.14, 0.06) | 0.444 |
|  | ln_MEP | 0.00 | (-0.04, 0.04) | 0.905 |  | 0.03 | (-0.02, 0.08) | 0.265 |  | 0.00 | (-0.04, 0.05) | 0.868 |  | -0.01 | (-0.05, 0.03) | 0.767 |  | **-0.08** | **(-0.14, -0.01)** | **0.028** |
|  | ln_MnBP | -0.01 | (-0.09, 0.08) | 0.905 |  | 0.00 | (-0.11, 0.11) | 0.943 |  | 0.06 | (-0.03, 0.15) | 0.211 |  | -0.07 | (-0.15, 0.02) | 0.137 |  | -0.10 | (-0.24, 0.05) | 0.210 |
|  | ln_MiBP | -0.04 | (-0.14, 0.06) | 0.399 |  | 0.06 | (-0.06, 0.19) | 0.294 |  | 0.00 | (-0.1, 0.11) | 0.937 |  | 0.00 | (-0.09, 0.10) | 0.933 |  | -0.01 | (-0.17, 0.16) | 0.943 |
|  | ln_MBzP | 0.00 | (-0.04, 0.05) | 0.845 |  | 0.00 | (-0.05, 0.06) | 0.898 |  | -0.01 | (-0.06, 0.04) | 0.733 |  | -0.02 | (-0.06, 0.03) | 0.405 |  | **-0.08** | **(-0.15, 0.00)** | **0.044** |
|  | ln_MEHP | -0.03 | (-0.08, 0.03) | 0.338 |  | 0.01 | (-0.06, 0.07) | 0.858 |  | 0.06 | (0.00, 0.11) | 0.057 |  | **-0.05** | **(-0.11, 0.00)** | **0.035** |  | 0.05 | (-0.03, 0.14) | 0.236 |
|  | **ln_MEHHP** | **-0.16** | **(-0.27, -0.06)** | **0.003** |  | -0.10 | (-0.24, 0.03) | 0.143 |  | 0.08 | (-0.04, 0.19) | 0.206 |  | **-0.15** | **(-0.25, -0.05)** | **0.003** |  | -0.09 | (-0.27, 0.08) | 0.297 |
|  | **ln_MEOHP** | **-0.10** | **(-0.20, 0.00)** | **0.048** |  | -0.06 | (-0.18, 0.07) | 0.352 |  | **0.12** | **(0.02, 0.23)** | **0.021** |  | **-0.17** | **(-0.26, -0.07)** | **0.001** |  | -0.07 | (-0.23, 0.09) | 0.402 |
|  | **ln_ΣDEHP**  **(μmol/ g creatinine)** | **-0.28** | **(-0.46, -0.1)** | **0.002** |  | -0.12 | (-0.33, 0.09) | 0.264 |  | 0.08 | (-0.11, 0.27) | 0.396 |  | -0.14 | (-0.29, 0.01) | 0.071 |  | 0.08 | (-0.19, 0.34) | 0.568 |
| Female | ln_MMP | -0.01 | (-0.07, 0.05) | 0.721 |  | 0.01 | (-0.08, 0.10) | 0.787 |  | 0.00 | (-0.09, 0.10) | 0.983 |  | 0.04 | (-0.03, 0.11) | 0.305 |  | -0.03 | (-0.15, 0.09) | 0.602 |
|  | ln_MEP | 0.00 | (-0.04, 0.04) | 0.917 |  | -0.02 | (-0.08, 0.03) | 0.407 |  | 0.00 | (-0.06, 0.06) | 0.920 |  | 0.01 | (-0.04, 0.05) | 0.811 |  | 0.06 | (-0.02, 0.13) | 0.137 |
|  | ln_MnBP | 0.06 | (-0.03, 0.14) | 0.178 |  | -0.05 | (-0.18, 0.09) | 0.494 |  | 0.09 | (-0.04, 0.23) | 0.174 |  | -0.04 | (-0.15, 0.07) | 0.457 |  | 0.01 | (-0.17, 0.18) | 0.941 |
|  | ln_MiBP | -0.02 | (-0.09, 0.06) | 0.659 |  | -0.08 | (-0.19, 0.03) | 0.138 |  | 0.07 | (-0.04, 0.18) | 0.220 |  | -0.01 | (-0.10, 0.08) | 0.802 |  | 0.05 | (-0.10, 0.19) | 0.512 |
|  | **ln_MBzP** | 0.02 | (-0.03, 0.06) | 0.466 |  | 0.03 | (-0.04, 0.09) | 0.449 |  | 0.00 | (-0.07, 0.07) | 0.970 |  | -0.01 | (-0.07, 0.04) | 0.655 |  | **-0.10** | **(-0.19, -0.01)** | **0.024** |
|  | **ln_MEHP** | -0.03 | (-0.08, 0.02) | 0.204 |  | 0.04 | (-0.04, 0.11) | 0.336 |  | -0.08 | (-0.15, 0.00) | 0.050 |  | **0.06** | **(0.00, 0.12)** | **0.038** |  | -0.04 | (-0.14, 0.05) | 0.360 |
|  | ln_MEHHP | 0.00 | (-0.10, 0.09) | 0.940 |  | -0.05 | (-0.19, 0.08) | 0.460 |  | -0.02 | (-0.16, 0.12) | 0.755 |  | -0.02 | (-0.13, 0.09) | 0.779 |  | -0.14 | (-0.32, 0.03) | 0.110 |
|  | ln_MEOHP | 0.00 | (-0.08, 0.09) | 0.976 |  | -0.07 | (-0.19, 0.06) | 0.307 |  | -0.01 | (-0.14, 0.12) | 0.835 |  | -0.02 | (-0.12, 0.09) | 0.750 |  | -0.15 | (-0.31, 0.02) | 0.078 |
|  | ln_ΣDEHP  (μmol/ g creatinine) | -0.02 | (-0.14, 0.10) | 0.777 |  | -0.10 | (-0.26, 0.06) | 0.239 |  | -0.07 | (-0.24, 0.10) | 0.409 |  | 0.02 | (-0.12, 0.16) | 0.759 |  | -0.06 | (-0.25, 0.13) | 0.525 |

ΣDEHP was estimated exposure of DEHP by summation of MEHP, MEHHP, and MEOHP exposure level. Adj-β, regression coefficient adjusted for maternal age at enrollment, maternal education status, household income, maternal exercise habit, and pre-pregnancy BMI. CI, confidence interval. *P*, p-value.
