# Supplementary material for: Prenatal phthalate exposure and sex steroid hormones in newborns: Taiwan Maternal and Infant Cohort Study
Source: PLoS One. 2024 Mar 14;19(3):e0297631. doi: 10.1371/journal.pone.0297631 (PMC10939196; doi:10.1371/journal.pone.0297631)
Supplement: S5 Table — (DOCX) [file pone.0297631.s008.docx]

**S5 Table. Associations of products of cord-blood sex hormones and maternal sex hormone concentrations with AGD in male and female newborns.**

| Steroid sex hormone levels | Original unit | Male newborns (n=141) | | |  | Female newborns (n=122) | | |
| --- | --- | --- | --- | --- | --- | --- | --- | --- |
|  |  | Adjusted β | (95% CI) | *P* |  | Adjusted β | (95% CI) | *P* |
| Cord-blood hormones |  |  |  |  |  |  |  |  |
| ln(P4) | ng/mL | -1.53 | (-4.23, 1.17) | 0.264 |  | 0.96 | (-0.91, 2.82) | 0.312 |
| ln(E2) | pg/mL | -0.59 | (-2.24, 1.06) | 0.480 |  | 0.03 | (-1.02, 1.09) | 0.951 |
| ln(frTT) | ng/mL | -0.54 | (-3.10, 2.02) | 0.675 |  | -0.63 | (-2.13, 0.86) | 0.400 |
| ln(SHBG) | nmol/L | -0.03 | (-1.99, 1.93) | 0.977 |  | 0.62 | (-0.62, 1.86) | 0.324 |
| ln(FSH) | mIU/mL | -0.58 | (-1.87, 0.71 | 0.376 |  | -0.45 | (-1.19, 0.28 | 0.229 |
| ln(E2$\boldsymbol{\times}$FSH) | pg/mL$\boldsymbol{\times}$mIU/mL | -0.61 | (-1.65, 0.43) | 0.245 |  | -0.44 | (-1.19, 0.30) | 0.244 |
| ln(P4$\boldsymbol{\times}$FSH) | ng/mL$\boldsymbol{\times}$mIU/mL | -0.72 | (-1.86, 0.41) | 0.210 |  | -0.28 | (-1.01, 0.44) | 0.438 |
| ln(frTT$\boldsymbol{\times}$LH) | ng/dL$\boldsymbol{\times}$mIU/mL | -0.44 | (-1.24, 0.36) | 0.281 |  | -0.90 | (-2.20, 0.39) | 0.169 |
| Maternal hormones |  |  |  |  |  |  |  |  |
| ln(P4) | ng/mL | -0.91 | (-3.3, 1.49) | 0.455 |  | 1.15 | (-0.41, 2.71) | 0.147 |
| ln(TT) | ng/dL | -0.32 | (-2.82, 2.18) | 0.800 |  | -0.48 | (-2.73, 1.76) | 0.672 |
| ln(E2) | pg/mL | -1.13 | (-3.84, 1.58) | 0.409 |  | 0.72 | (-1.03, 2.46) | 0.418 |

E2, estradiol. FSH, follicle stimulating hormone. P4, progesterone. TT, total testosterone. LH, luteinizing hormone.

Adjusted β, regression coefficient adjusted for maternal age at enrollment and maternal education status. CI, confidence interval. *P*, p-value.
